# Viscoelasticity in Large Deformation Analysis of Hyperelastic Structures

**DOI:** 10.3390/ma15238425

**Published:** 2022-11-26

**Authors:** Shahriar Dastjerdi, Bekir Akgöz, Ömer Civalek

**Affiliations:** 1 Division of Mechanics, Civil Engineering Department, Akdeniz University, Antalya 07070, Turkey; 2 Department of Medical Research, China Medical University Hospital, China Medical University, Taichung 404, Taiwan

**Keywords:** nonlinear hyperelastic material, viscoelasticity, semi-analytical polynomial method, large deformation

## Abstract

In this paper, an annular/circular plate made of hyperelastic material and considering the viscoelastic property was investigated based on a novel nonlinear elasticity theory. A new approach for hyperelastic materials in conjunction with the Kelvin–Voigt scheme is employed to obtain the structure’s large deformation under uniform transverse loading. The constitutive equations were extracted using the energy method. The derived partial differential time-dependent equations have been solved via the semi-analytical polynomial method (SAPM). The obtained results have been validated by ABAQUS software and the available paper. In consequence, a good agreement between the results was observed. Finally, several affecting parameters on the analysis have been attended to and studied, such as the nonlinear elasticity analysis, the boundary conditions, loading, and the material’s viscosity. It can be possible to obtain the needed time for achieving the final deformation of the structure based on the applied analysis in this research.

## 1. Introduction

Various materials can be found in nature that demonstrate different mechanical behaviors under applied loads, such as metals displaying linear elastic behavior until yielding. At the same time, irreversible deformation is then observed up to failure. On the other hand, brittle materials exhibit a slight elastic deformation linearly and then fail without irreversible deformation. However, it is notable that all materials do not exhibit linear elasticity. Hyperelastic materials will exhibit elastic deformations excessively before failure without irreversible deformation. These materials exhibit an extremely nonlinear stress-strain behavior that ascends monotonically up to fracture. It is well-known that linear elastic materials can be defined with two material constants: modulus of elasticity and Poisson’s ratio. On the contrary, hyperelastic materials can be defined by a strain-energy density function. The stored strain energy remains constant when a sample is subjected to constant strain. Therefore, hyperelastic materials are modeled in the context of strain energy potentials such as Arruda-Boyce [1], Gent [2], Neo-Hookean [3], and Yeoh [4].

Some recent studies on the mechanical responses of hyperelastic structures are summarized below. Erchiqui et al. [5] implemented a dynamic finite element method to model the visco-hyperelastic behaviors of a thin, isotropic, and incompressible thermoplastic membrane based on the Ogden and Mooney-Rivlin models. Kocatürk and Akbaş [6] examined the geometrically nonlinear static response of a hyperelastic simply supported beam under a non-follower load by the finite element and Newton–Raphson iteration methods. Li et al. [7] perused the dynamic behavior of the visco-hyperelastic dielectric elastomer structures based on the Gent hyperelastic model. Alibakhshi et al. [8] analyzed the nonlinear vibration of dielectric elastomer microbeam resonators based on a hyperelastic Cosserat model by the Runge-Kutta time domain method. Almasi et al. [9] studied the thermomechanical analysis of a hyperelastic thick cylindrical pressure vessel by giving analytical and numerical solutions. Asgari and Hashemi [10] developed an efficient visco-hyperelastic constitutive model for a hollow cylinder elastomer under dynamic and impact loadings. Pascon [11,12] implemented a finite element formulation based on a two-dimensional beam element to investigate various viscoelastic functionally graded materials and beams made of functionally graded hyperelastic material. The constitutive equations are extracted based on the neo-Hookean model. Gharooni and Ghannad [13] studied the nonlinear analysis of functionally graded tapered hyperelastic cylindrical pressure vessels subjected to non-uniform pressure load.

Hosseini and Rahimi [14] conducted the nonlinear bending analysis of a neo-Hookean hyperelastic plate based on the Mindlin plate theory. Xu et al. [15] introduced the plate element formulation by quadratic interpolation. They carried out static and dynamic analyses of incompressible hyperelastic silicone plates. Dadgar-Rad and Firouzi [16] presented a nonlinear finite element formulation for the viscoelastic deformation of hyperelastic structures under several loadings and boundary conditions. Ansari et al. [17] developed a numerical approach to survey the deformations of hyperelastic Mindlin rectangular plates in compressible and nearly incompressible regimes based on the Neo-Hookean model. Tashiro et al. [18] analyzed blood clots using a nonlinear viscoelastic and hyperelastic model. They employed the visco-hyperelastic finite element method to estimate the mechanical behavior of blood clots. Shariyat and Abadi [19] studied the nonlinear dynamic and impact responses of incompressible neo-Hookean hyperelastic plates with stiff elastic reinforcing particles. Runge-Kutta time integration and penalty methods are used in the solution. Karimi et al. [20] conducted the nonlinear dynamic analysis of an embedded neo-Hookean hyperelastic membrane under a uniformly distributed hydrostatic pressure. Alibakhshi and Heidari [21] carried out the nonlinear vibration of a dielectric elastomer balloon via the Gent hyperelastic model. A time integration-based solver is utilized to solve the resulting equations. Alibakhshi et al. [22] elaborated on a suitable detection mechanism for scanning the surface profile of a micro-sample by AFM, considering that the probe is made of a hyperelastic material. Falope et al. [23] proposed a finite element-based theoretical model to study the bending behavior of a hyperelastic solid.

Hosseini et al. [24] perused the nonlinear static behavior of functionally graded hyperelastic plates based on FSDT. The potential energy function is formulated according to the neo-Hookean model and the Cauchy–Green tensor. Coda et al. [25] elaborated a finite element formulation to analyze the laminated and functionally graded hyperelastic one-dimensional structure with transverse shear stress distribution. Dastjerdi et al. [26] introduced a comprehensive theoretical method to examine the mechanical responses of hyperelastic structures. The proposed method can analyze the geometrically and physically nonlinear hyperelastic materials. Zhao et al. [27] investigated the nonlinear dynamic responses of the visco-hyperelastic spherical shells under uniform radial loads. Additionally, Zhao et al. [28] perused dynamic loads and structural damping influences for incompressible hyperelastic spherical shells. They considered the Yeoh strain energy function in their study. Bacciocchi and Tarantino [29] examined the finite bending of hyperelastic beams based on the compressible Mooney-Rivlin model. More information about the nonlinear dynamics of hyperelastic structures can be found in the recent review article [30]. Additionally, many studies have been carried out on analyzing viscoelastic structures [31,32,33,34,35,36,37,38,39,40,41,42,43,44,45]. Li et al. [46] presented a perturbation approach for the lateral vibration analysis of viscoelastic microstructures. The clamped microbeam has been subjected to external harmonic excitation. Recently, some researchers developed a structure-preserving approach to solve the macroscopic/microscopic coupling dynamic problems with large deformation [47]. Additionally, a large deformation analysis of a nano-sized structure has been attended by Yan et al. [48].

As stated before, many recent studies have been performed on the mechanical analysis of structures made of hyperelastic materials based on various strain energy functions, especially the neo-Hookean model. However, there is no study on the static response of an annular circular visco-hyperelastic plate. The new approach for hyperelastic materials simulates the annular circular visco-hyperelastic plate in conjunction with the Kelvin–Voigt scheme. The general formulations are derived according to the first-order shear deformation theory (FSDT). The constitutive equations and boundary conditions are extracted by the energy method. Then, the semi-analytical polynomial method is utilized for solving the partial differential time-dependent equations. Finally, the effects of various parameters on the bending analysis of the annular circular visco-hyperelastic plate are investigated and discussed in detail.

## 2. Geometry of the Structure

**Figure 1:**
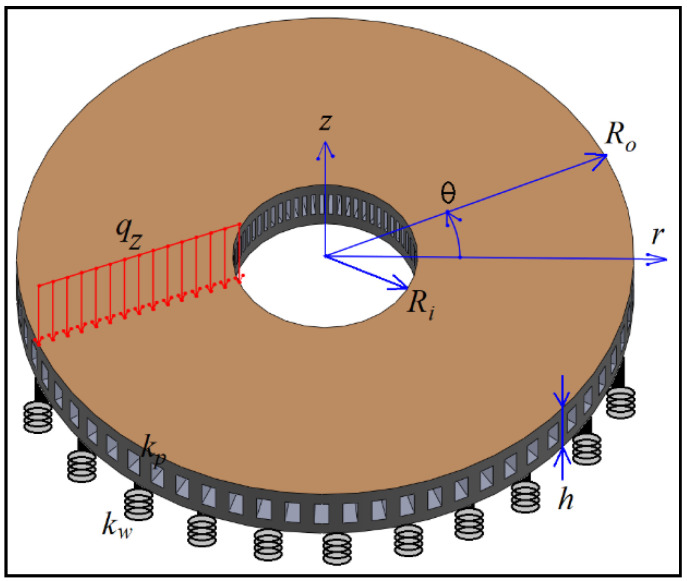
Schematic view of the structure.

First, the geometry of the problem is discussed. The geometry of the analyzed structure will play a significant role in obtaining the governing equations. The more comprehensive the geometry of the problem, the more different structures can be modeled only by considering a single formulation. Analysis of the structures made of hyperelastic materials will make the simulation more complicated. Therefore, an attempt has been made to focus more on the effect of viscosity on hyperelastic materials. Hence, an annular–circular structure made of visco-hyperelastic material has been assumed. The geometry of the analyzed problem is shown in Figure 1, along with the considered coordinate system, which is the cylindrical coordinate system.

As can be seen, the inner radius of the structure is $R_{i}$, and its outer radius is $R_{o}$. Additionally, the thickness of the structure is constant and equal to h. The structure is placed on an elastic foundation with two components, the Winkler ($k_{w}$) and Pasternak ($k_{p}$). The external load on the structure is considered a uniform distributed load in the z direction equal to $q_{z}$.

## 3. Viscoelastic Property

**Figure 2:**
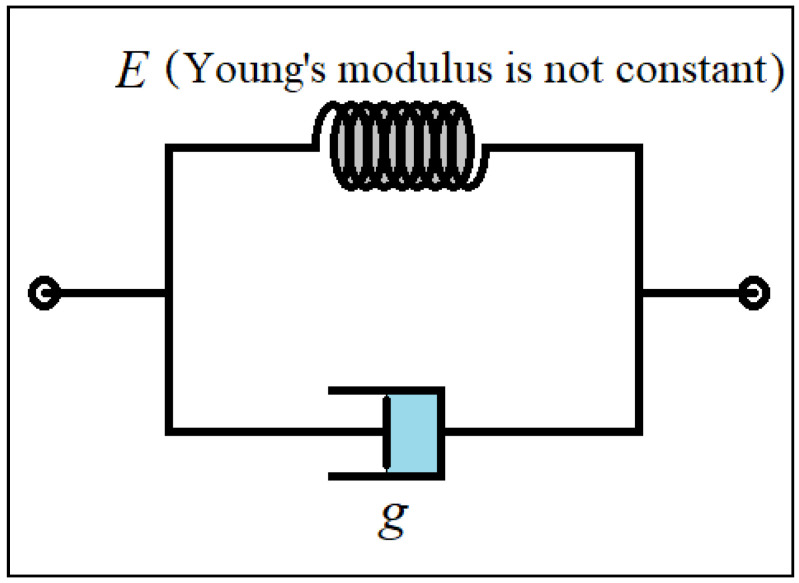
The Kelvin–Voigt model for viscoelastic property.

In this research, an attempt has been made to perform two nonlinear elastic properties simultaneously with the changes in the deformation of the material with respect to the strain rate. In other words, large strains against the strain rate are considered. The structure’s material is considered in such a way that the deformation created in it due to the applied load is different during the duration of the load application. On the other hand, the final deformation of the structure does not happen as soon as the load is applied, and it takes place over time. The Kelvin–Voigt model is used to consider the viscoelastic properties of the material [43]. Additionally, other models such as Zener and mixing linear viscoelasticity and nonlinear elasticity (as has been done in this paper) have been used by other researchers [49,50]. In this type of time-dependent modeling for stress–strain, a spring and a damper are placed in parallel, which can be seen in Figure 2. As shown in Figure 2, the viscous property of the material (time-dependent strain) is simulated by a damper. Additionally, the material’s elasticity is modeled by a spring (E represents Young’s elasticity modulus). In linear elastic materials, the value of E is constant and changes linearly. However, this value is not fixed or variable in this research. Therefore, the model (as explained in the next part) uses linear viscoelasticity according to the Kelvin–Voigt scheme. However, the principle of elasticity has been considered nonlinearly.

Stress and strain will be written according to the following equations based on the Kelvin–Voigt simulation.

(1) $$\sigma_{Total}=\sigma_{s}+\sigma_{D}\left(\sigma_{s}=E\varepsilon \left(r,\theta ,z,t\right),\sigma_{D}=g\frac{\partial \varepsilon \left(r,\theta ,z,t\right)}{\partial t}\right)$$

It can be seen in Figure 2 that E is the elasticity modulus, and g is the viscosity of the material. The amount of $\sigma_{s}$ is linear here according to Equation (1). However, it is not constant in this study due to the nonlinear elasticity analysis. This issue will be discussed in detail in the next part.

## 4. Nonlinear Elastic Material and Governing Equations

In a linear elastic material, it can be observed that the changes in stress and strain are linear. However, in a structure with nonlinear elastic property, the stress increases nonlinearly with the increase of strain. In this research, the material with nonlinear elastic properties is assumed. Therefore, calculations based on nonlinear elasticity theory are considered. Various mathematical simulations and theories have been presented regarding the analysis of nonlinear elastic or hyperelastic structures [2,9,10,11,12]. A new method has been presented previously for analyzing the mechanical behavior of nonlinear elastic structures [26]. According to this theory, the stress-strain diagram of a nonlinear elastic material obtained from the experiment is approximated by a polynomial function ($\sigma \left(\varepsilon \right)=\overset{n}{\underset{i=1}{\sum }}E_{i}\varepsilon^{i}$). According to the polynomial degree, a higher accuracy can be obtained by choosing more n. Consequently, the expressed viscous stress can be formulated as $\sigma_{s}\left(\varepsilon \right)=\overset{n}{\underset{i=1}{\sum }}E_{i}\varepsilon {\left(r,\theta ,z,t\right)}^{i}$. The calculations related to the strain field and the governing equations can be obtained according to the following equations by considering the viscoelastic property.

$${\overset{↔}{\varepsilon }}_{ij}=\left[\begin{array}{ccc} \varepsilon_{rr} & \varepsilon_{r\theta } & \varepsilon_{rz}\\ \varepsilon_{\theta r} & \varepsilon_{\theta \theta } & \varepsilon_{\theta z}\\ \varepsilon_{zr} & \varepsilon_{z\theta } & \varepsilon_{zz} \end{array}\right]$$

(2) $$\begin{array}{ll} \varepsilon_{rr}=\left(1+g\frac{\partial }{\partial t}\right) & \left(\frac{\partial U_{r}}{\partial r}+\frac{1}{2}{\left(\frac{\partial U_{z}}{\partial r}\right)}^{2}\right);2\varepsilon_{r\theta }=2\varepsilon_{\theta r}=0;\;2\varepsilon_{rz}=2\varepsilon_{zr}\\ & =\left(1+g\frac{\partial }{\partial t}\right)\left(\frac{\partial U_{r}}{\partial z}+\frac{\partial U_{z}}{\partial r}\right);\;\varepsilon_{\theta \theta }\\ & =\left(1+g\frac{\partial }{\partial t}\right)\left(\frac{1}{r}\left(\frac{\partial U_{\theta }}{\partial \theta }+U_{r}\right)\right);\;2\varepsilon_{\theta z}=2\varepsilon_{z\theta }=0;\;\varepsilon_{zz}=0 \end{array}$$

The strain field is assumed based on FSDT as

(3) $$\{\begin{array}{l} U_{r}\left(r,z,t\right)=u_{0}\left(r,t\right)+z\varphi \left(r,t\right)\\ U_{\theta }\left(r,z\right)=0\\ U_{z}\left(r,z,t\right)=w_{0}\left(r,t\right) \end{array}$$

$$\sigma_{rr}=\overset{n}{\underset{i=1}{\sum }}\frac{E_{i}}{{\left(1-\nu^{2}\right)}^{i}}{\left[\varepsilon_{rr}+\nu \varepsilon_{\theta \theta }\right]}^{i};\sigma_{\theta \theta }=\overset{n}{\underset{i=1}{\sum }}\frac{E_{i}}{{\left(1-\nu^{2}\right)}^{i}}{\left[\varepsilon_{\theta \theta }+\nu \varepsilon_{rr}\right]}^{i};$$

(4) $$\sigma_{rz}=\overset{n}{\underset{i=1}{\sum }}\frac{E_{i}}{{\left[2\left(1+\nu \right)\right]}^{i}}\varepsilon_{rz}^{i}$$

In the above equations, $u_{0}$ and $w_{0}$ are transport displacements, and φ is the rotation function around the θ axes. Now, using the energy method [43,44,45] ($\delta P_{Total}={∭}_{V}\sigma_{ij}\delta \varepsilon_{ij}+\iint_{A}\left(q_{z}-k_{W}w_{0}+k_{p}\nabla^{2}w_{0}\right)\delta w_{0}\;\left(i,j=r,\theta ,z\right)$), the mathematical description of boundary conditions and governing equations can be introduced as the following equations.

(5) $$\delta u_{0}:\frac{\partial N_{rr}}{\partial r}+\frac{1}{r}\left(N_{rr}-N_{\theta \theta }\right)=0$$

(6) $$\delta w_{0}:\frac{\partial N_{rz}}{\partial r}+\frac{N_{rz}}{r}+N_{rr}\frac{\partial^{2}w_{0}}{\partial r^{2}}+\frac{N_{rr}}{r}\frac{\partial w_{0}}{\partial r}+\frac{\partial N_{rr}}{\partial r}\frac{\partial w_{0}}{\partial r}+q_{z}-k_{w}w_{0}+k_{p}\nabla^{2}\left(w_{0}\right)=0$$

(7) $$\delta \varphi :\frac{\partial M_{rr}}{\partial r}+\frac{1}{r}\left(M_{rr}-M_{\theta \theta }\right)-N_{rz}=0$$

(8) $$\left(N_{rr},N_{\theta \theta },N_{rz}\right)=\int_{-\frac{h}{2}}^{\frac{h}{2}}\left(\sigma_{rr},\sigma_{\theta \theta },\sigma_{rz}\right)dz;\;\left(M_{rr},M_{\theta \theta }\right)=\int_{-\frac{h}{2}}^{\frac{h}{2}}\left(\sigma_{rr},\sigma_{\theta \theta },\right)zdz$$

Considering the uniform transverse load on the structure, the problem is symmetrical, and there will be no changes in the θ direction. The only independent variables of the problem are r and t, where t represents the time duration. The mathematical definition of the different types of boundary conditions is introduced according to the following relations: Clamped (C), Simply supported (S), and Free (F) at the edges of $r=R_{i},\;R_{o}$.

(9) $$\{\begin{array}{l} Clamped:\;u_{0}=w_{0}=\varphi =0\;\left(r=R_{i},\;R_{o};t=0,t_{N}\right)\\ Simply\;supported:\;u_{0}=w_{0}=M_{rr}=0\;\left(r=R_{i},\;R_{o};t=0,t_{N}\right)\\ Free:\;N_{rr}=N_{rz}=M_{rr}=0\;\left(r=R_{i},\;R_{o};t=0,t_{N}\right) \end{array}$$

## 5. Solution Method

This research uses the semi-analytical method based on polynomials (SAPM) to solve the governing equations [43,44]. According to this method, the displacement functions ($u_{0},w_{0}$, and φ) are formulated as comprehensive polynomials based on the number of nodes (N and M) in each direction of the independent variable of the problem (r and z).

(10) $$u_{0}\left(r,z\right)=\overset{N}{\underset{i=1}{\sum }}\overset{M}{\underset{j=1}{\sum }}a_{\left(j+M\left(i-1\right)\right)}r^{\left(i-1\right)}t^{\left(j-1\right)}$$

(11) $$w_{0}\left(r,z\right)=\overset{N}{\underset{i=1}{\sum }}\overset{M}{\underset{j=1}{\sum }}a_{\left(j+M\left(i-1\right)+M\cdot N\right)}r^{\left(i-1\right)}t^{\left(j-1\right)}$$

(12) $$\varphi \left(r,z\right)=\overset{N}{\underset{i=1}{\sum }}\overset{M}{\underset{j=1}{\sum }}a_{\left(j+M\left(i-1\right)+2M\cdot N\right)}r^{\left(i-1\right)}t^{\left(j-1\right)}$$

Now, a system of algebraic equations can be obtained by inserting the functions (Equations (10)–(12)) in the mathematical definition of the boundary conditions at the edges and constitutive equations. Finally, by solving the obtained algebraic equations, the unknown functions in the displacement field and, accordingly, other unknowns, including stresses and strains, will be obtained for the visco-hyperelastic annular–circular sheet under uniform transverse load.

## 6. Discussion

### 6.1. Validation

#### 6.1.1. The Solving Method (SAPM)

**Figure 3:**
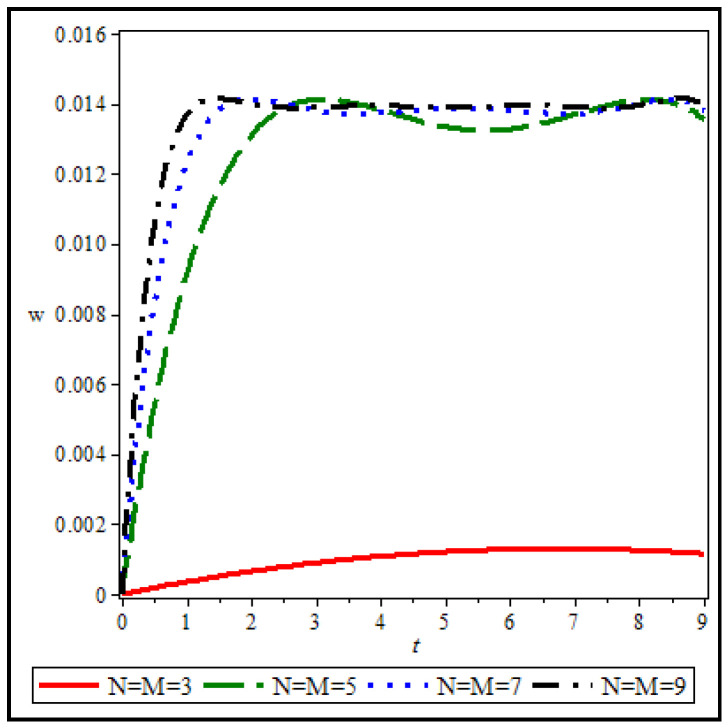
Investigation of the result’s convergence.

First, the accuracy of the used solution method is checked. Figure 3 shows the background changes in terms of time for an annular/circular sheet with the following specifications for different values of the number of nodes in the direction of r and t.

(13) $$R_{i}=0.1m,R_{o}=0.5m,h=0.03m,E_{1}=2.1467\times 10^{6},E_{2}=-6.4659\times 10^{5},E_{3}\;\;=-69779,E_{4}=5.66989\times 10^{5},\nu =0.3,q_{z}=1000Pa,$$

As can be seen, the accuracy of the results increases versus the increase of nodes in the calculations. A sudden jump is observed between the results of N=M=3 and N=M=5, so the used solution method has a very high convergence. It can be seen that the results of seven and nine nodes are very close, and therefore, the results obtained from seven or nine nodes can be used with reasonable confidence. Increasing the number of nodes in the calculations will significantly increase the time to obtain the results. Therefore, the optimized number of nodes in the network for solving the problem is an important point that should be considered. In the first form, by choosing only nine nodes in each direction (r and t), you can get the results with the desired accuracy for the subsequent analysis.

#### 6.1.2. Comparisons

**Figure 4:**
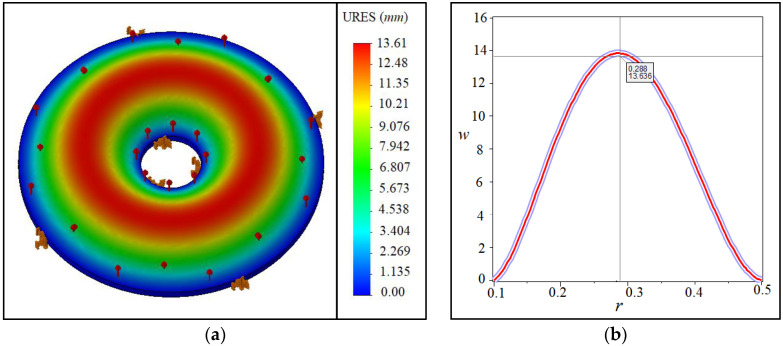
Deflection results of (**a**) ABAQUS software and (**b**) the present study.

**Figure 5:**
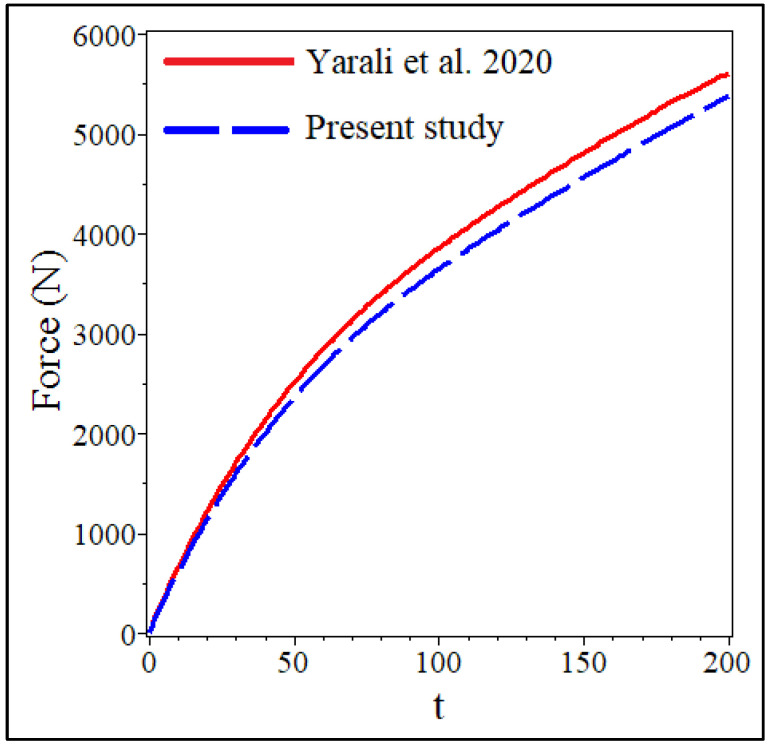
Comparison between the results of this study and [51] for the visco-hyperelastic analysis.

Figure 4a,b and Table 1 are provided to validate the results. Figure 4 shows the maximum deflection between the results of (a) the present study and (b) the obtained results of ABAQUS software for an annular/circular plate. It is demonstrated that the results agree well. Finding relevant papers in visco-hyperelasticity to fit with the present work is hard. For example, some papers can be found. However, their modeled geometry might be different from the present study. Another assessment was done, and the results can be observed in Figure 5 for the visco-hyperelastic structure. As the tested model [51] is a thick circular sheet, we considered a quasi-three-dimensional analysis [52]. Therefore, the captured results have been monitored into a single plot in conjunction with the experimental results [51]. The compared results in Figure 5 show that an acceptable difference is available between the obtained results. The obtained results in this paper are near to the ABAQUS results, according to the originally depicted figure in [51]. Eventually, the applied theory and solving method are reliable. Additionally, according to Table 1, the same conclusion can be made for different boundary conditions.

### 6.2. Numerical Results

**Figure 6:**
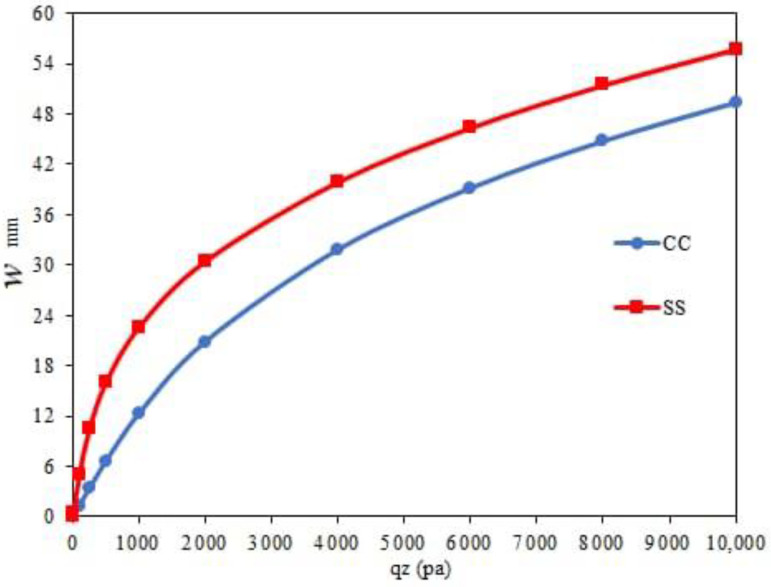
Deflection changes versus the applied transverse loading ($q_{z}$) for different types of boundary conditions.

To investigate different boundary conditions on nonlinear-elastic analysis, Figure 6 depicts a circular sheet with the same characteristics as Figure 3. As can be seen, the maximum deflection increases with the increase of transverse load on the structure. The increase in the maximum deflection is completely nonlinear and will be accompanied by a decreasing slope. In other words, as the transverse load on the sheet increases, it is observed that the slope of the changes decreases. The importance of this matter is that if the linear analysis were considered, the obtained results would increase linearly. These linear results would be acceptable only for low load values. However, because the nonlinear analysis of the sheet made of material with nonlinear elastic properties has been studied in this research, the simulation results can be used with appropriate accuracy. Another point (according to Figure 6, which is drawn for two boundary conditions, CC and SS, at the edges of $R_{i}$ and $R_{o}$) is that the decreasing trend of the results at the beginning of loading is more for the CC boundary conditions than SS. However, with increasing load on the plate, the changes are almost the same, with a slight difference for the two boundary conditions, CC and SS. In other words, by increasing the load on the structure, the effects related to the boundary conditions are reduced, and the behavior of the sheet against the applied load will be less affected by the applied boundary conditions.

**Figure 7:**
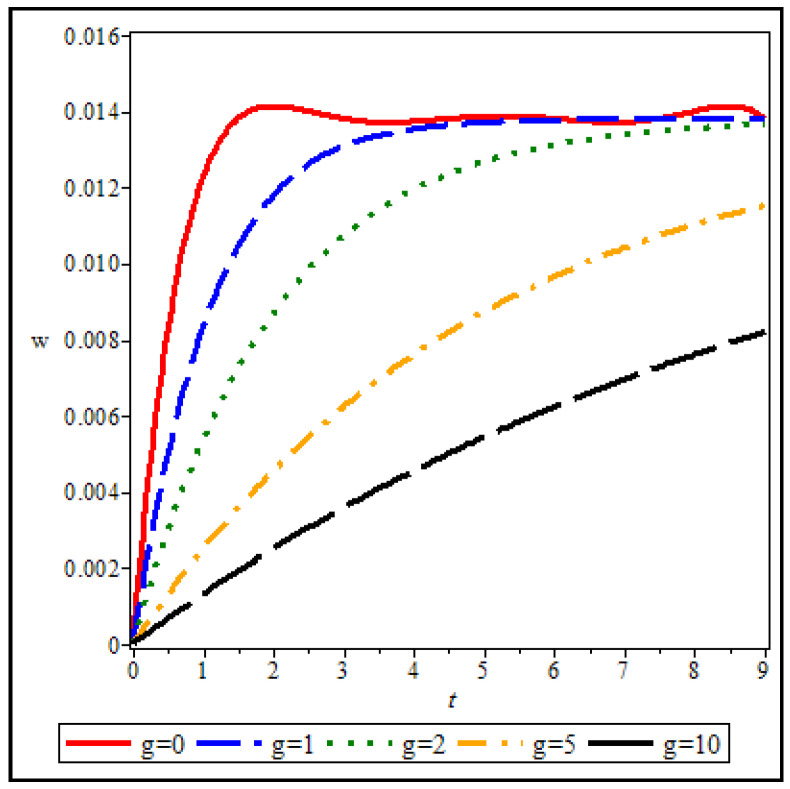
Viscoelastic effects on the results.

**Figure 8:**
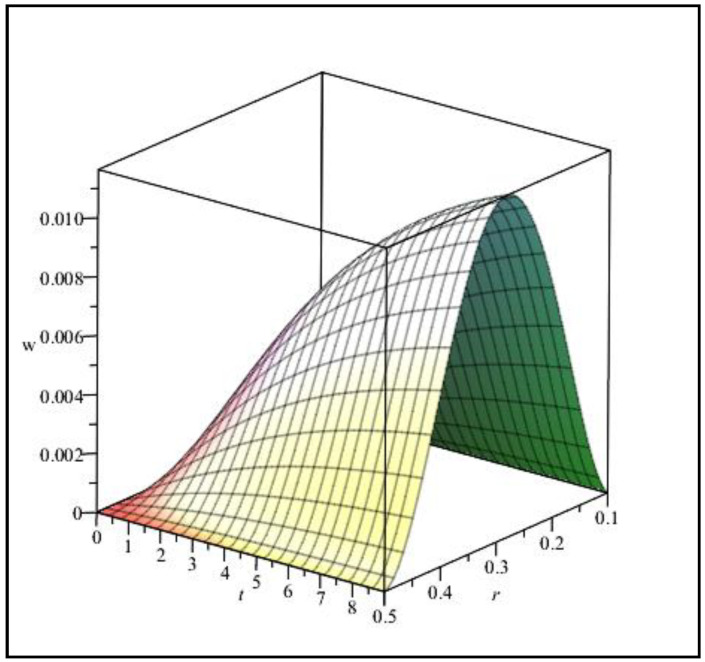
Three-dimensional view of deflection changes versus r and time (t) for g=5.

The viscoelastic property in nonlinear elasticity analysis is a significant issue considered in this research. Therefore, Figure 7 and Figure 8 show the effect of viscosity (changes in deformation over time) on the results of the maximum deflection of the circular sheet (specifications of Figure 3). Viscosity g=0 means that, when the load is applied on the sheet, the maximum deflection will occur, and the structure will reach the absolute limit of deformation, which can be seen in Figure 7. Of course, it can be observed that the mentioned result will be for t=1s. This calculation error is due to the limitation in choosing the number of nodes in time when solving the governing equations. It can be observed that with the rise of the viscosity value (g), the needed time for the sheet to reach its final deformation will increase. In other words, with the increase in viscosity, the slope variations will decrease over time. Of course, the effect of viscosity on the results is not linear. In other words, the distance between the results from g=0 to g=5 is more remarkable than from g=5 to g=10. As the viscosity of the structure material increases, the intensity of its impact on the results decreases. Figure 8 is one of the results of the curves in Figure 7 (g=5), which here show the changes in the deflection in two directions: r and t, simultaneously. According to Figure 8, the maximum deflection on the sheet will occur in its middle ($r=R_{i},\;R_{o}$), according to the CC boundary conditions.

**Figure 9:**
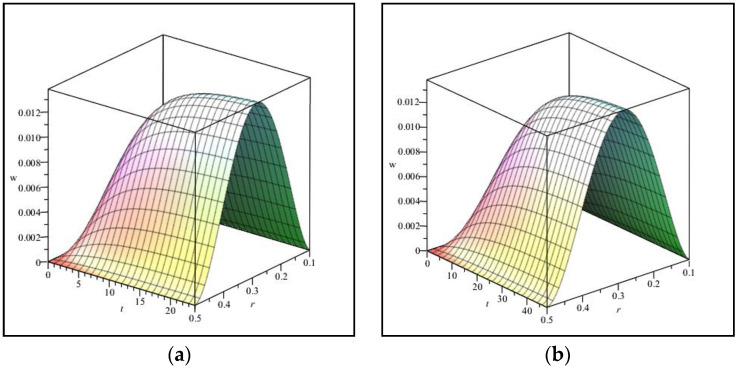
Deflection changes versus r and time for (**a**) g=5 and (**b**) g=10.

In Figure 7 and Figure 8, the effect of viscosity on the nonlinear elastic structure is investigated. As seen, with the increase in viscosity, the time for the structure to reach its final shape increases. Now, this topic, how much time is required for the final deflection (considering a specific viscosity), is further investigated. Figure 9a is drawn for viscosity g=5, and Figure 9b is drawn for g=10. Here, the criterion for drawing two shapes is to reach the absolute limit of the deformation and be equal to a specific number. In other words, the final deflection for both Figure 9a,b is equal. It can be seen that the final deflection in Figure 9a is for t=25s. However, this result in Figure 9b is equal to t=50s. This analysis is crucial, because it is observed that, with the doubling of the viscosity of the structure, the time it takes to reach the final shape change also doubles. Although the variation rate is nonlinear, the deformation of the structure at the final time has a linear and direct relationship with the value of the viscosity of the structure. Of course, this conclusion has been reached for this particular problem. Such a result may not be obtained by considering other assumptions, including environmental factors such as temperature, humidity, or electric fields. Investigating the impact of the mentioned factors can also be considered by other researchers who work in this field.

## 7. Conclusions

The present study investigated the viscoelastic analysis of nonlinear elastic (hyperelastic) materials. The governing partial differential equations and mathematical definitions of boundary conditions were derived based on a new method and were solved by the semi-analytical method based on polynomials (SAPM). The viscoelastic property of the structural material is assumed by Kelvin–Voigt modeling. In general, the significant results obtained from the research can be categorized as follows:

1. The new theory of hyperelastic structures can be used with appropriate confidence for viscoelastic properties.
2. Structures made of nonlinear elastic material are sensitive to changes in applied transverse loads, and the changes are entirely nonlinear, even with the low-load application.
3. For low loads, it has a significant impact on the deformation by the boundary conditions. However, as the load increases, these effects decrease.
4. As the viscosity increases, the duration of the final deformation increases, which has a direct relationship with the viscosity of the material.

## Figures and Tables

**Table 1:** Comparison between the maximum deflection (mm) results of the present study and [53] for aluminum circular plates.

Maximum Deflection (mm)								
Boundary Conditions	$q_{z}\;=\;5\;MPa$		$q_{z}\;=\;20\;MPa$		$q_{z}\;=\;50\;MPa$		$q_{z}\;=\;100\;MPa$	
	Present	[53]	Present	[53]	Present	[53]	Present	[53]
SS	3.789	3.883	8.232	8.333	12.11	12.15	15.56	15.68
CC	1.261	1.263	4.559	4.562	8.819	8.820	12.93	12.93

## Data Availability

There is no data for sharing, and all data are available within the paper.
